# Short-term cytotoxic effects and long-term instability of RNAi delivered using lentiviral vectors

**DOI:** 10.1186/1471-2199-5-9

**Published:** 2004-08-03

**Authors:** Richard J Fish, Egbert KO Kruithof

**Affiliations:** 1 Division of Angiology and Haemostasis, Department of Internal Medicine, Geneva University Hospital and University Medical Centre, Geneva, CH-1211 Switzerland

## Abstract

**Background:**

RNA interference (RNAi) can potently reduce target gene expression in mammalian cells and is in wide use for loss-of-function studies. Several recent reports have demonstrated that short double-stranded RNAs (dsRNAs), used to mediate RNAi, can also induce an interferon-based response resulting in changes in the expression of many interferon-responsive genes. Off-target gene silencing has also been described, bringing into question the validity of certain RNAi-based approaches for studying gene function. We have targeted the plasminogen activator inhibitor-2 (PAI-2 or SERPINB2) mRNA using lentiviral vectors for delivery of U6 promoter-driven PAI-2-targeted short hairpin RNA (shRNA) expression. PAI-2 is reported to have anti-apoptotic activity, thus reduction of endogenous expression may be expected to make cells more sensitive to programmed cell death.

**Results:**

As expected, we encountered a cytotoxic phenotype when targeting the PAI-2 mRNA with vector-derived shRNA. However, this predicted phenotype was a potent non-specific effect of shRNA expression, as functional overexpression of the target protein failed to rescue the phenotype. By decreasing the shRNA length or modifying its sequence we maintained PAI-2 silencing and reduced, but did not eliminate, cytotoxicity. ShRNA of 21 complementary nucleotides (21 mers) or more increased expression of the oligoadenylate synthase-1 (OAS1) interferon-responsive gene. 19 mer shRNA had no effect on OAS1 expression but long-term selective pressure on cell growth was observed. By lowering lentiviral vector titre we were able to reduce both expression of shRNA and induction of OAS1, without a major impact on the efficacy of gene silencing.

**Conclusions:**

Our data demonstrate a rapid cytotoxic effect of shRNAs expressed in human tumor cell lines. There appears to be a cut-off of 21 complementary nucleotides below which there is no interferon response while target gene silencing is maintained. Cytotoxicity or OAS1 induction could be reduced by changing shRNA sequence or vector titre, but stable gene silencing could not be maintained in extended cell culture despite persistent marker gene expression from the RNAi-inducing transgene cassette. These results underscore the necessity of careful controls for immediate and long-term RNAi use in mammalian cell systems.

## Background

Gene silencing is a powerful tool with which to study protein function. Gene inactivations in mice have revolutionised the way we study both basic biology and a plethora of disease types [1,2]. Gene silencing in human cells has, until recently, proven difficult to achieve [3]. Research with plants, flies and worms recently uncovered a mechanism by which eukaryotic cells target mRNAs, and perhaps even genetic loci, for specific gene silencing. This process is termed RNA interference (RNAi). RNAi can also be induced in mammalian cells using double-stranded RNAs (dsRNAs), and has become the method of choice for targeted knock-down of gene expression in mammalian cells [4]. The apparent specificity of RNAi also enables allele-specific gene targeting [5]. Initial studies using RNAi in mammalian cells centred around transient knock-down of target gene expression, either using direct transfection of synthetic short interfering RNA (siRNA) [6], transfection of in vitro transcribed siRNA [7] or transient expression of short dsRNA via transfection of plasmid DNA bearing RNA Polymerase III promoter-driven expression cassettes [8,9]. Short dsRNAs of 19 to 29 base-paired nucleotides, complementary to the target mRNA, were expressed as 2 complementary RNAs or as a hairpin structure (shRNA), and resulted in knock-down of the target message. While these initial RNAi methods gave a rapid phenotypic read-out in vitro, stable knock-down of gene expression is required for monitoring long-term effects on cell function, for example, in developing tumors in vivo or in cell-based gene therapy approaches. Efficient delivery of RNAi-inducing dsRNA or expression cassettes is required for effective transient and long-term studies. Transfer of functional shRNAs using lentiviral vectors appears to be a valid approach for effective, stable construct delivery to both cell lines [10] and primary cells [11-13]. More recently, using several different expression systems and target cells, reports have highlighted the utility and specificity of the RNAi approach [14-17].

Maintaining RNAi-inducing dsRNA below 30 nucleotides in length was thought to avoid activation of the interferon-induced anti-viral response. PKR is a key anti-viral regulator and its expression can be induced by the interferon response [18]. PKR is activated when bound to dsRNA longer than 30 nucleotides, despite interacting with shorter dsRNA molecules [19]. Four recent reports have pointed towards limitations to using RNAi as a tool in mammalian cells. The first demonstrated off-target gene silencing [20], highlighting the redundancy of short nucleotide sequences in the human transcriptome. The second characterised the expression profile of genes as a result of lentiviral vector-mediated RNAi. Interferon regulated gene expression was increased even with dsRNAs as short as 19 nucleotides [21]. The third report demonstrated similar interferon response gene up-regulation, after transfection of cell lines with synthetic siRNAs as short as 21 nucleotides [22]. Finally, Scacheri et al documented significant siRNA sequence-dependent changes in the expression of non-targeted proteins [23].

In this work we used a simple approach for gene silencing in human tumor cell lines, using lentiviral vectors for stable delivery of shRNAs. We aimed to study the effects of targeting the plasminogen activator inhibitor-2 (PAI-2 or SERPINB2) mRNA on cell survival in the presence of pro-apoptotic stimuli. In addition to its inhibitory activity on the urokinase plasminogen activator, PAI-2 is thought to have anti-apoptotic properties [24,25]. Its molecular targets in this respect are unknown. A recent report demonstrated a functional interaction between PAI-2 and the retinoblastoma protein cell cycle regulator [26].

Using lentiviral vectors for delivery of RNAi-inducing expression cassettes we achieved potent PAI-2 gene silencing, accompanied by a rapid cytotoxic effect. The degree of cytotoxicity was proportional to shRNA length and induction of an interferon response gene could be detected when shRNA of 21 complementary base pairs or more was expressed. The phenotype was not target gene specific, as PAI-2 overexpression failed to rescue cytotoxicity and control hairpins were also cytotoxic. Using lower vector titre, reduced shRNA expression and interferon response induction was measured without compromising gene silencing. Using a 19 complementary base pair shRNA expression vector, which reduced PAI-2 expression and induced no initial cytotoxicity or interferon response, transduced cell marker gene expression was maintained but gene silencing lost in long-term cell culture. Our results highlight the need for careful controls to monitor specificity and maintenance of gene silencing when using RNAi for stable loss-of-function studies in mammalian cells.

## Results

### Efficient transfer of RNAi-inducing cassettes using lentiviral vectors

**Figure 1:**
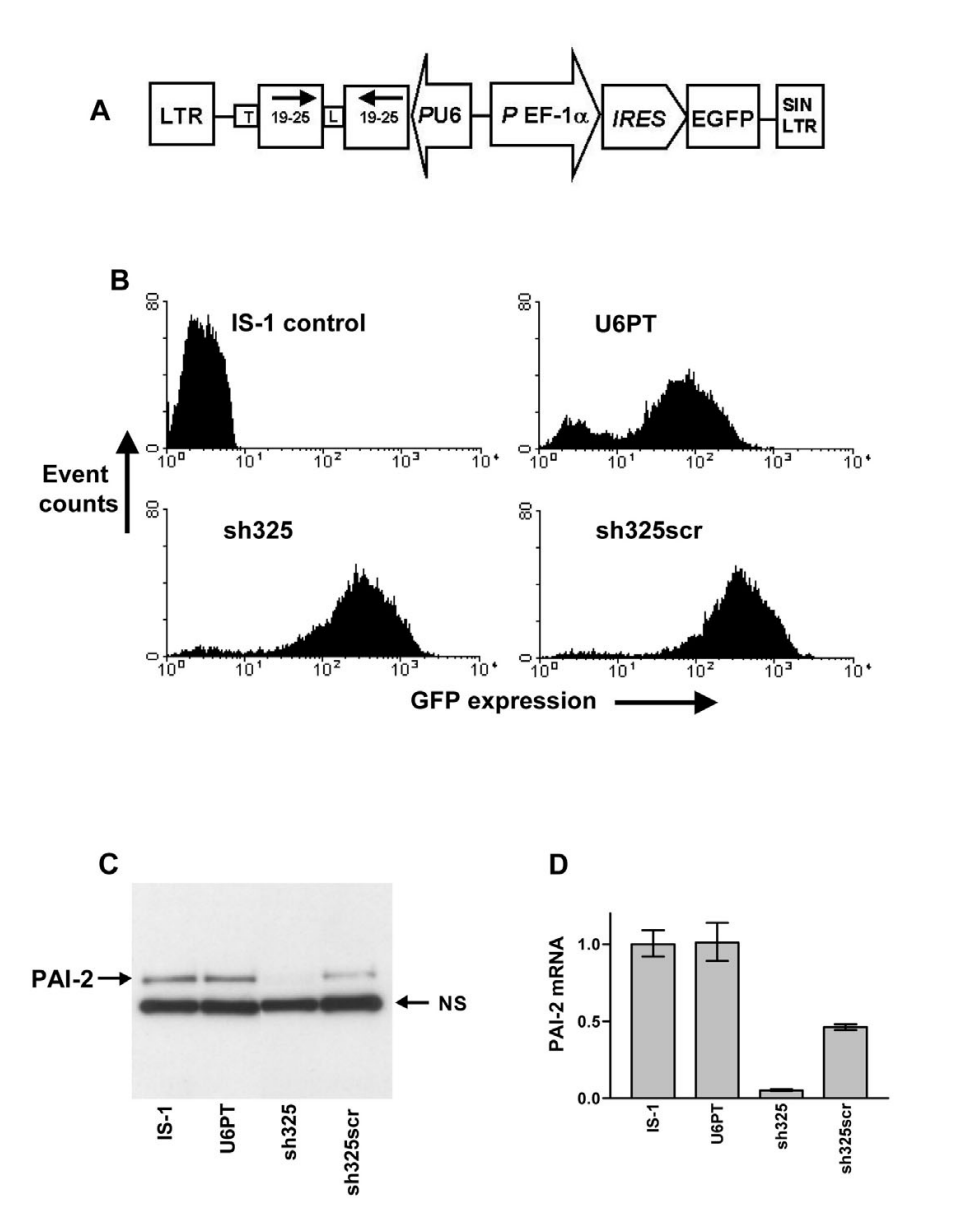
**Effective gene silencing using lentiviral vectors for RNAi. **The gene transfer cassette common to each vector for RNAi is shown in A. Each construction for RNAi was designed for expression of a shRNA, homologous to the target mRNA or with a scrambled sequence, driven by the RNA polymerase III-controlled human U6 promoter and ending with a terminator (T) sequence. The shRNA is represented by two arrows which encode 19 to 25 nucleotide complementary sequences and are joined by an eight nucleotide loop (L). EGFP expression is via the EF-1α promoter, oriented in the opposite direction, driving an IRES sequence and the EGFP gene. Each cassette is flanked by the HIV long terminal repeats (LTR), of which the 3' LTR is modified to ensure that the vectors are self-inactivating upon integration (SIN). B shows flow cytometry analysis of non-transduced IS-1 cells and cells four days after transduction with the U6PT control vector, a vector for expression of shRNA complementary to a region of the PAI-2 mRNA (sh325) and a vector for expression of a shRNA with a scrambled sh325 sequence (sh325scr). C shows an immunoblot for detection of PAI-2 in the cell lysate of these cells. NS highlights a single non-specific band which is consistently detected in PAI-2 immunoblots using IS-1 cell lysates. In D, PAI-2 mRNA levels from the same samples are measured by QRT-PCR of cDNA, using the ΔCT method and hypoxanthine phosphoribosyl transferase (HPRT) as the control gene. Each target gene was detected in duplicate, error bars represent the standard deviation of mean values.

Lentiviral vectors were generated which deliver an expression cassette for human U6 promoter-driven expression of short hairpin RNA (shRNA), with exact homology to the human PAI-2 mRNA. The vector expression cassette also bears the enhanced green fluorescent protein (GFP) gene under the control of the EF-1α promoter, and an internal ribosome entry site (IRES) sequence (see Figure 1A). This cassette allows permanent expression of GFP in transduced cells, and the possibility of concomitant overexpression of a further cDNA, between the EF-1α promoter and IRES sequences, not used here. The shRNA sequences were chosen from the PAI-2 mRNA to include a 5' guanosine at the U6 promoter transcriptional start site, to exclude the 5' and 3' 100 nucleotides of the PAI-2 open reading frame, and to be between 30 and 70 % guanosine/cytidine rich. As controls, we have used a vector leading to expression of GFP alone (EGFP), a vector with the U6 promoter and transcriptional termination signals but lacking a hairpin encoding sequence (U6PT), and vectors leading to expression of scrambled sequences of certain hairpins. Figure 1 demonstrates the efficient transduction of Isreco-1 (IS-1) human colorectal carcinoma cells with one such PAI-2 targeting vector (sh325). Sh325 is designed for expression of a shRNA with a 25 nucleotide double-stranded stretch (a 25 mer) to target the PAI-2 mRNA. As controls, we used U6PT and a scrambled sequence (sh325scr) vector. Four days after transduction, each cell population expressed high levels of GFP, as a marker for transduction (Figure 1B). Compared to non-transduced cells, or cells transduced with the U6PT control vector, we measured a clear knock-down of endogenous PAI-2 protein and mRNA in cells transduced with the sh325 vector (see figure 1C and 1D).

However, the control vector with a scrambled sequence (sh325scr) also reduced PAI-2 mRNA and protein levels. Equal sample loading for immunoblots was confirmed by Ponceau S staining of nitrocellulose membranes (data not shown) and the presence of equal amounts of a PAI-2 monoclonal antibody-reactive non-specific band in each blot (NS in Figure 1C).

### Rapid cytotoxic effect of RNAi vectors

**Figure 2:**
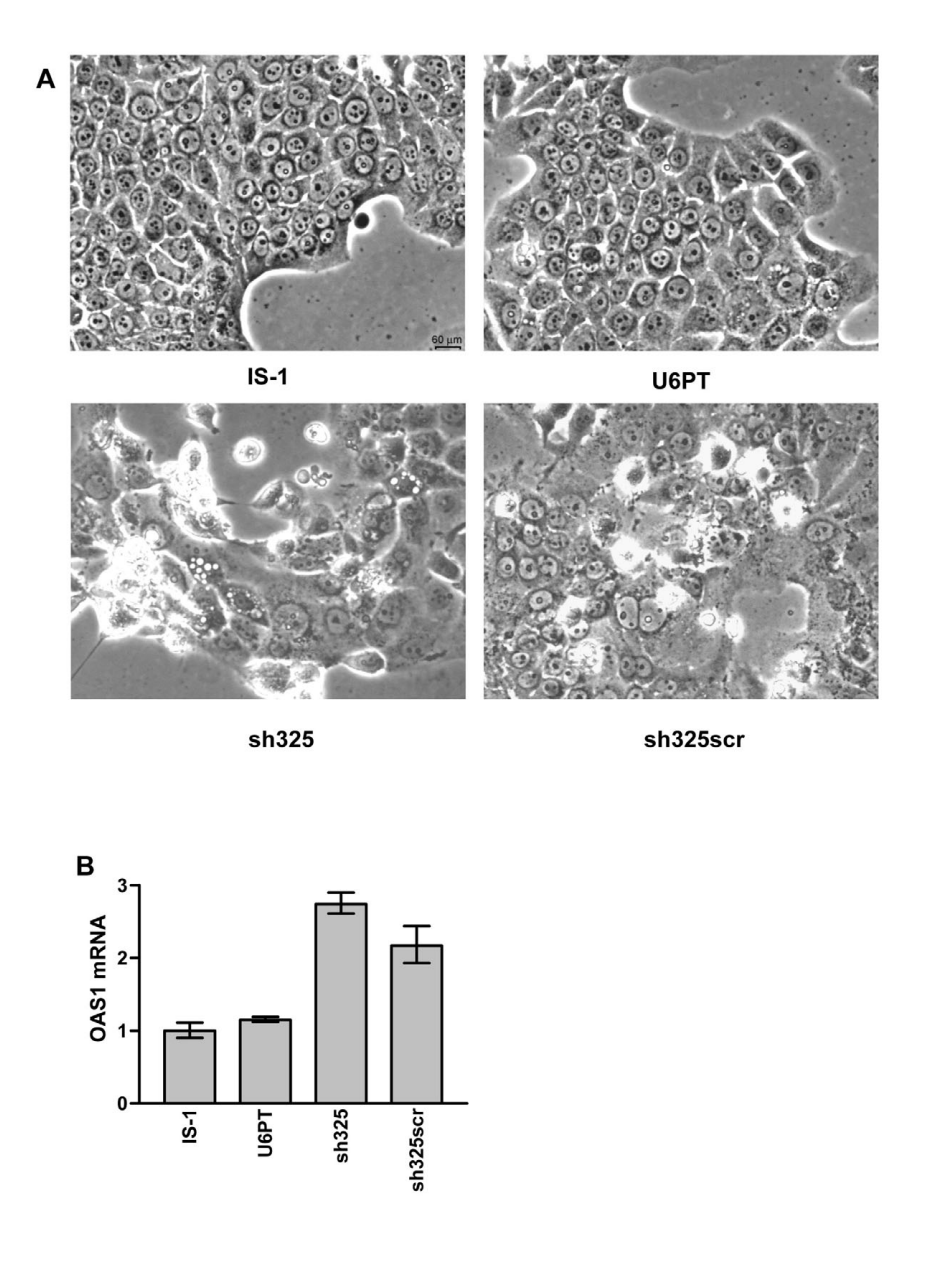
**Cytotoxicity and OAS1 induction with RNAi vectors. **In A, morphology was observed using phase contrast microscopy of non-transduced IS-1 cells, or cells six days after transduction with vectors leading to expression of no shRNA (U6PT), a 25 mer shRNA targeting PAI-2 (sh325) and a scrambled 25 mer control shRNA (sh325scr). B shows comparison of OAS1 expression in non-transduced cells or cells four days after transduction with U6PT, sh325 and sh325scr vectors, by QRT-PCR. Each target gene was detected in duplicate, error bars represent the standard deviation of mean values.

Many of the initial loss-of-function studies using RNAi have measured the phenotypic effect of gene silencing in the immediate time frame after introduction of the siRNA or RNAi-inducing expression vector. As seen in Figure 1, four days after transduction with our vectors appears to be sufficient for efficient target gene silencing. Transduction with lentiviral vectors leads to stable long-term integration of the desired transgene cassette, a key advantage in their use compared to other transient or less stable expression systems. Thus we reasoned that transduction with RNAi-inducing cassettes, using lentiviral vectors, would also be stable unless the reduction in target gene expression gave transduced cells a significant growth disadvantage or cytotoxic phenotype. Four to five days after transduction, IS-1 cells bearing the sh325 construct or cells transduced with a scrambled sh325 sequence rapidly changed morphology, compared to U6PT-transduced control cells. Sh325-transduced cells began to disintegrate into small particles and detach from cell culture dishes. After 10 days most of the sh325-transduced cells were dead while the U6PT-transduced cells were growing like the parent cell line. The scrambled hairpin vector-transduced cells gave a weaker cytotoxic phenotype, with deteriorating cell morphology and some detachment of transduced cells. Figure 2A shows the morphology of IS-1 cells 6 days after transduction. To understand further this cytotoxic effect, we performed quantitative RT-PCR (QRT-PCR) on RNA isolated from IS-1 cells, 4 days after transduction with the same vectors, in order to measure the levels of the 2'5'-oligoadenylate synthetase-1 (OAS1) mRNA after transduction with each vector. The OAS1 gene is recognised as an interferon response gene and has also been monitored elsewhere when using RNAi [21]. We measured increases in OAS1 expression in both sh325 and scrambled sh325 vector-transduced cells, whereas control-transduced cells (U6PT) had equal OAS1 levels to non-transduced cells (see Figure 2B).

### 19 mer shRNAs induce less cytotoxicity than longer hairpins and do not increase OAS1 expression

**Figure 3:**
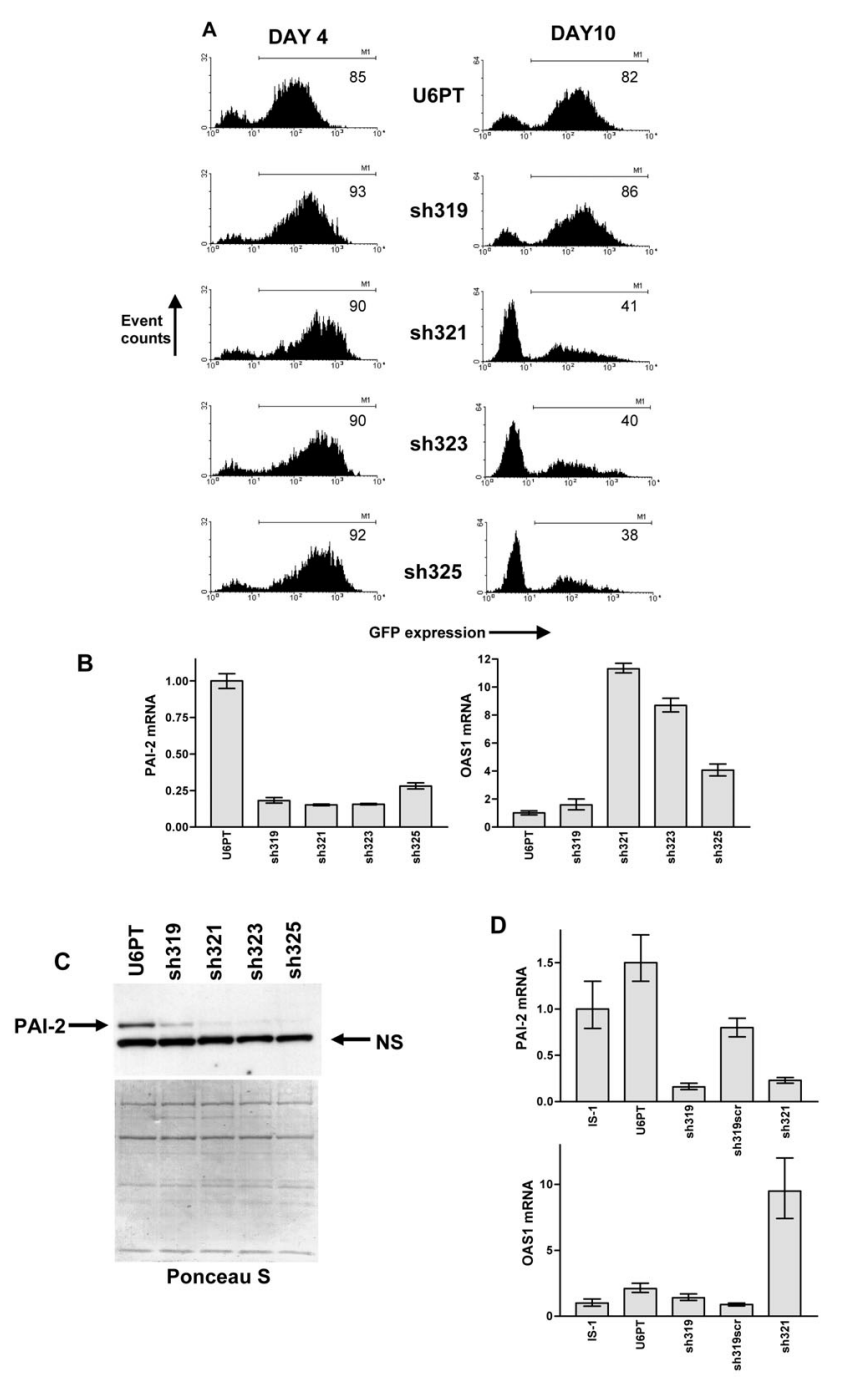
**Shorter shRNA length reduced, but did not eliminate, cytotoxicity. **A represents flow cytometry analysis of IS-1 cells 4 and 10 days after transduction with U6PT, sh319, sh321, sh323 and sh325 vectors. GFP expression is detected, and the percentage of GFP expressing cells was determined using the M1 gating shown (percentage GFP positive cells is shown in each histogram). B shows a comparative analysis of PAI-2 and OAS1 mRNA, in the samples described in A, 4 days after transduction. Data were generated by QRT-PCR and error bars are as described in previous figures. In C, cell lysates from samples of transduced cells described in A and B were subjected to immunoblotting with anti-PAI-2 monoclonal antibodies. Ponceau S staining served as a gel loading control, as did comparison of a single non-specific band (NS) in the immunoblot. D shows QRT-PCR analysis, as in B, for IS-1 cell mRNAs after transduction with or without U6PT, sh319, sh319scr and sh321 vectors.

As both the target gene-specific and the scrambled sequence 25 mer shRNAs, sh325 and sh325scr, induced the OAS1 interferon response gene, we generated further vectors for delivery of shRNAs with reduced hairpin length. We reduced the length of sh325, from 25 to 23, 21 and 19 nucleotides and named the novel vectors sh323, sh321 and sh319, respectively. The truncations were made at the 3' end of the 25 nucleotide sense strand and therefore the 5' of its complementary anti-sense sequence (see Table 1). Each vector was used to transduce IS-1 cells and the growth of GFP positive cells monitored at 4 and 10 days after transduction, compared to U6PT control-transduced cells (see Figure 3A). Targeting of the PAI-2 mRNA and protein was monitored, four days after transduction, by QRT-PCR and immunoblotting of cell lysates (Figure 3B and 3C). While each PAI-2 targeted vector successfully reduced PAI-2 mRNA and protein four days after transduction, a strong negative selection was seen for shRNA-expressing cells after a further six days of culture. This selective pressure on transduced cells was stronger with the 21 mer, 23 mer and 25 mer shRNAs than with the shorter sh319-derived 19 mer (Figure 3A). OAS1 mRNA levels were measured by QRT-PCR of transduced cell cDNA four days after transduction (Figure 3B). The cells transduced with the 21 mer, 23 mer and 25 mer shRNAs showed induction of OAS1 mRNA, however, contrary to our expectations, highest OAS1 levels were obtained with the 21 mer shRNA. Loss of GFP positive cells over time was comparable for 21 mer, 23 mer and 25 mer hairpin constructs.

To determine whether the lack of OAS1 induction was specific to sh319 or common to other 19 mer shRNAs, further transductions and QRT-PCR analysis were performed on mRNA from IS-1 cells transduced with sh319, sh321 and sh319scr vectors. sh319scr encodes a shRNA with a scrambled sh319 sequence. This analysis also confirms the specificity of the PAI-2 silencing, by comparing sh319 to sh319scr. Figure 3D shows that no induction of OAS1 was measured using sh319 or sh319scr vectors and sh319scr had no effect on the PAI-2 mRNA level.

**Table 1:** Construct details and shRNA sequences. Vector names and the shRNA sequences they encode. In comments, numbers are coding human PAI-2 mRNA nucleotides (adapted from accession number M18082).

	Hairpin sequence			
Name	sense	loop	antisense	Comments

sh319	GCGCACACCUGUACAGAUG	CAAGCUUC	CAUCUGUACAGGUGUGCGC	PAI-2 684–702
sh321	GCGCACACCUGUACAGAUGAU	CAAGCUUC	AUCAUCUGUACAGGUGUGCGC	PAI-2 684–704
sh323	GCGCACACCUGUACAGAUGAUGU	CAAGCUUC	ACAUCAUCUGUACAGGUGUGCGC	PAI-2 684–706
sh325	GCGCACACCUGUACAGAUGAUGUAC	CAAGCUUC	GUACAUCAUCUGUACAGGUGUGCGC	PAI-2 684–708
sh319scr	GUCAUACCGGCAAGGAUCC	CAAGCUUC	GGAUCCUUGCCGGUAUGAC	scrambled sh319
sh325scr	GGCCGGAGAUAAGUUCACUCAACUC	CAAGCUUC	GAGUUGAGUGAACUUAUCUCCGGCC	scrambled sh325
sh119	GAAGACCAGAUGGCCAAGG	CAAGCUUC	CCUUGGCCAUCUGGUCUUC	PAI-2 151–169
EGFP	No hairpin, empty vector.			none
U6PT	Human U6 promoter/terminator, no hairpin.			none

### Cytotoxicity is not target gene specific

**Figure 4:**
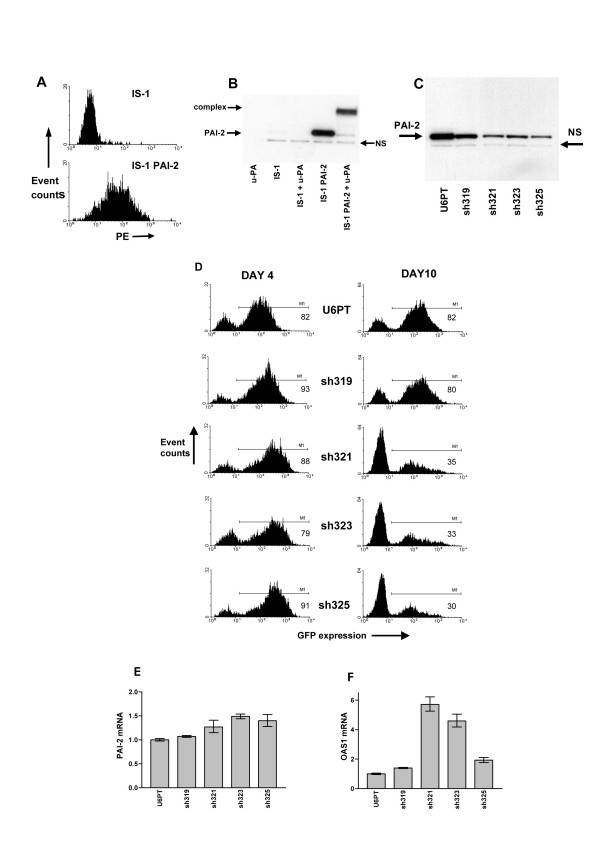
**PAI-2-targeted RNAi with overexpression of functional PAI-2 in IS-1 cells. **IS-1 cells were transduced with a lentiviral vector for delivery of the human PAI-2 cDNA under control of the CMV promoter. A shows a flow cytometry analysis for detection of PAI-2 in transduced IS-1 PAI-2, and non-transduced IS-1 cells. Both cell types were fixed, permeabilised and labelled with anti-PAI-2 monoclonal antibodies, then incubated with PE-labelled secondary antibodies. In B, the functional activity of overexpressed PAI-2 was assessed by immunoblotting of cell lysates, for PAI-2 expression, with or without the addition of 10U of u-PA. u-PA alone is included in lane 1. In C, PAI-2 protein levels were assessed in cell lysates from IS-1 PAI-2 cells, after transduction with U6PT, sh319, sh321, sh323 and sh325 vectors. NS in B and C highlights a single non-specific band which is consistently detected in PAI-2 immunoblots of IS-1 cell lysates. D represents flow cytometry analysis of IS-1 PAI-2 cells 4 and 10 days after transduction with U6PT, sh319, sh321, sh323 and sh325 vectors. GFP expression is detected, and the percentage of GFP expressing cells was determined using the M1 gating shown (percentage GFP positive cells are given in each histogram). E and F show comparative analyses of PAI-2 and OAS1 mRNA expression, respectively, in samples described in D. Samples were analysed 4 days after transduction. Data were generated by QRT-PCR, each target gene was detected in duplicate, error bars represent the standard deviation of mean values.

To determine if all or part of the cytotoxic effect seen with our shRNAs was due to down-regulation of PAI-2, we generated an IS-1 cell line which overexpresses functional PAI-2. A lentiviral vector was produced which delivers the wild type PAI-2 cDNA, and used to transduce IS-1 cells. This resulted in a homogeneous population of cells which overexpress PAI-2 (see Figure 4A, IS-1 PAI-2 cells). Using immunoblotting of PAI-2/u-PA complexes, formed by mixing IS-1 PAI-2 cell lysates with low molecular weight u-PA, we demonstrated that this overexpressed protein was functional (see Figure 4B). We transduced these cells with the series of shRNA-delivering vectors described in Figure 3 (sh325, sh323, sh321 and sh319), to test whether functional PAI-2 overexpression could reverse the cytotoxic phenotype. As even endogenous PAI-2 is not completely silenced using these vectors we reasoned that the RNAi they deliver would not be capable of functionally silencing overexpressed PAI-2. As predicted, our PAI-2 targeting shRNAs were unable to completely reduce the overexpressed PAI-2 protein levels (see Figure 4C, compared to the relative non-specific band intensity in Figure 3C). However, the cytotoxic effect seen with the parent IS-1 cell line was also clearly apparent in the PAI-2 overexpressing cells. We monitored the loss of GFP positive cells in the transduced PAI-2 overexpressing cell populations and saw almost identical kinetics, compared to the parent cell line (compare Figure 3A and Figure 4D). These data show that the cytotoxic effect is not target gene specific. We also measured the level of PAI-2 mRNA in this experiment, by QRT-PCR. Despite a several-fold decrease in overexpressed PAI-2 protein level (see immunoblot in Figure 4C) we were unable to detect knock-down of the overexpressed mRNA (Figure 4E). In a similar manner to the IS-1 parent cell line, transduction of PAI-2 overexpressing cells with sh325, sh323, sh321, but not the sh319 vector, induced the OAS1 interferon response gene (Figure 4F). To exclude IS-1 cell-specific effects of our vectors we transduced IS-1 cells and HeLa cells with a GFP control, the sh319 and the sh319 scrambled sequence (sh319scr) vectors. We monitored the percentage of GFP positive cells at day 4, 8 and 11 after transduction and observed similar selective loss of GFP positive cells for the sh319 and sh319scr vectors, in both cell lines (Table 2).

**Table 2:** Percentage GFP positive cells over time in shRNA-expressing IS-1 and HeLa cells. Transduced cells were assessed for GFP expression by flow cytometry. GFP positive cells were gated equally for each cell type, 4, 8 and 11 days after transduction with EGFP, sh319 and sh319scr vectors.

	% GFP positive cells					
	
	IS-1			HeLa		
	
Vector	Day 4	Day 8	Day 11	Day 4	Day 8	Day 11
	
EGFP	95	95	93	92	94	93
sh319	86	70	56	79	68	50
sh319scr	90	76	59	82	64	45

### Reducing lentiviral vector titre can reduce shRNA expression level and OAS1 induction, while maintaining gene silencing

**Figure 5:**
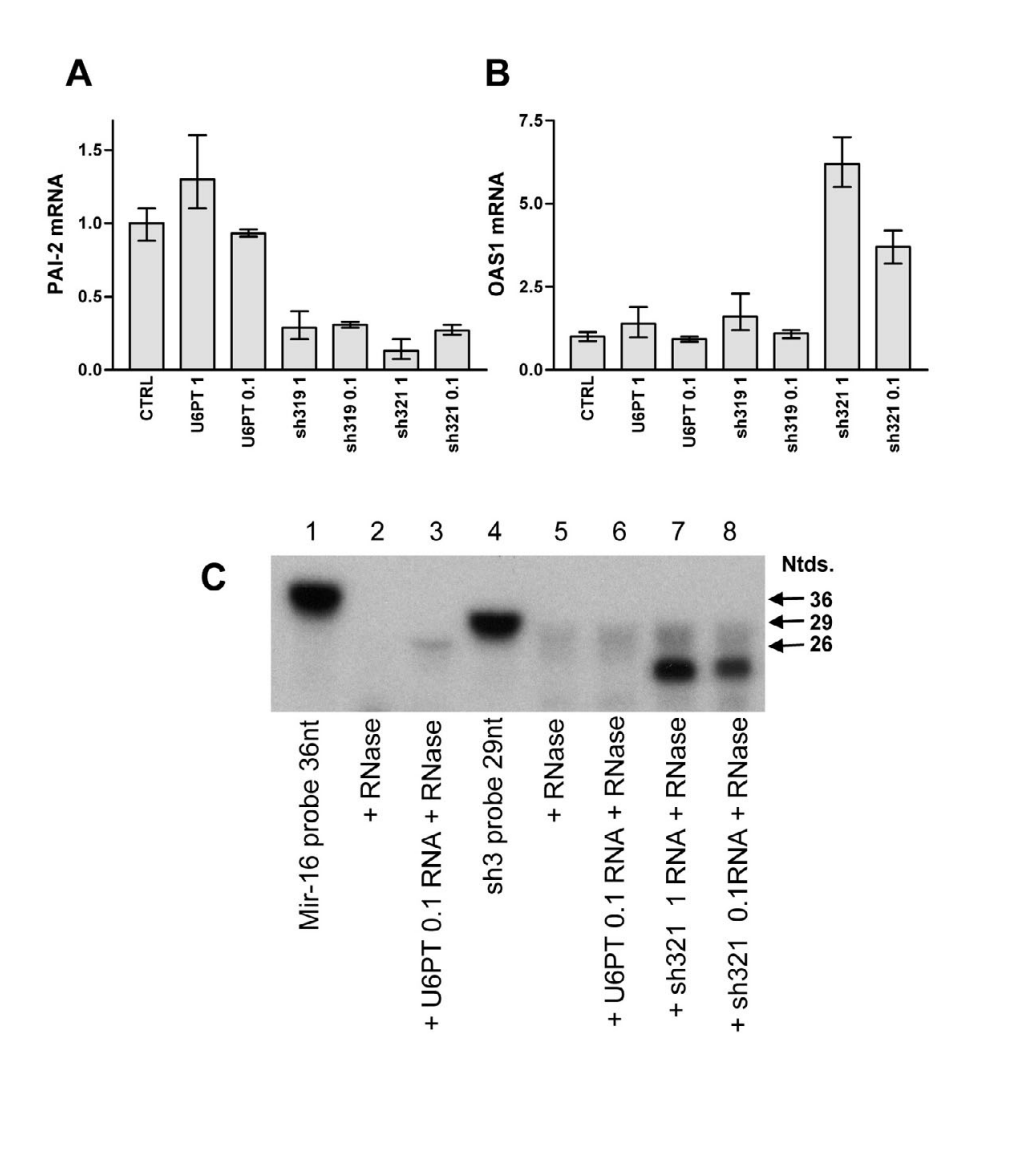
**Efficient target gene silencing with reduced OAS1 induction and lower shRNA expression. **Total RNA was isolated from IS-1 cells (CTRL) or IS-1 cells 4 days after transduction with U6PT, sh319 or sh321 vectors. After reverse transcription, cDNA was analysed for expression of PAI-2 (A) and OAS1 (B). Data were generated by QRT-PCR, each target gene was detected in triplicate, error bars represent the standard deviation of mean values. 1 ml or 0.1 ml of each lentiviral vector stock was used, hence the designations 1 and 0.1. Vector titres were approximately 10^6 ^transducing units per ml resulting in a multiplicity of transduction of approximately 10 for 1 ml used or 1 for 0.1 ml used. In C, shRNA expression was detected in total cell RNAs using a modified RNase protection protocol. Total RNA was mixed with radiolabelled probes for hybridization and RNase protection. Samples were resolved on a 15 % Acrylamide/8M Urea/TBE gel and RNase protected probes detected by autoradiography. Lane 1 shows the Mir-16 probe without RNase digestion or target RNA, lane 2 is as lane 1 with RNase digestion and lane 3 as lane 2 with U6PT-transduced cell RNA as hybridization target. Lane 4 shows the sh3 probe without RNase digestion or target RNA, lane 5 as lane 4 with RNase digestion and lane 6 as lane 5 with U6PT-transduced cell RNA as hybridization target. In lane 7 sh321-transduced cell RNA was used as the hybridization target for the sh3 probe with RNase digestion, and lane 8 is as lane 7 except that cells were transduced with 10-fold less vector titre (sh321 1 and sh321 0.1). Known nucleotide lengths (Ntds.) for the probes and the protected Mir-16 endogenous RNA are marked.

To understand whether lentiviral vector titre and resulting shRNA expression levels influence the non-specific effects described, IS-1 cells were transduced with the U6PT, sh319 and sh321 vectors as described above and also using a 10-fold reduction in vector titre (vector titre details are given in the figure 5 legend). Both sh319 and sh321 vectors effectively down-regulate PAI-2, but only the sh321 vector induces OAS1 expression (see Figure 3B). Using 10-fold lower vector titre had little impact on PAI-2 mRNA silencing, as seen using QRT-PCR (Figure 5A), and resulted in a small decrease in OAS1 mRNA induction (Figure 5B) in sh321-transduced cells. To assess whether lower vector titre resulted in lower shRNA expression, total RNA from cells transduced with different titres of sh321 vector was subjected to RNase digestion after hybridization with a ^32^-P labelled RNA probe, designed to protect the first 19 nucleotides of the sh325 shRNA. Upon hybridization, this "sh3" probe should protect short RNA expressed in sh321 vector-transduced cells from RNase digestion. As negative controls, RNA from U6PT-transduced cells was subjected to the RNase protection procedure, and the sh3 probe was treated with RNase without target RNA. As a positive control a probe for the Mir-16 miRNA was constructed and used to detect endogenous Mir-16 miRNA in U6PT-transduced cells using the same protocol (Figure 5C). Sh3 probe-protected RNA was detected in sh321-transduced cells. Reducing the viral titre clearly reduced the expression of shRNA and this correlated with reduced OAS1 induction. Sh3 probe-protected RNA was not detected in control-transduced cells and the sh3 probe was completely digested in the absence of target RNA (Figure 5C). This data demonstrates that vector-derived shRNA expression can be reduced without impacting gene silencing and that lower expression correlates with a reduced interferon response.

### Loss of long-term gene silencing despite persistent transduction marker gene expression

**Figure 6:**
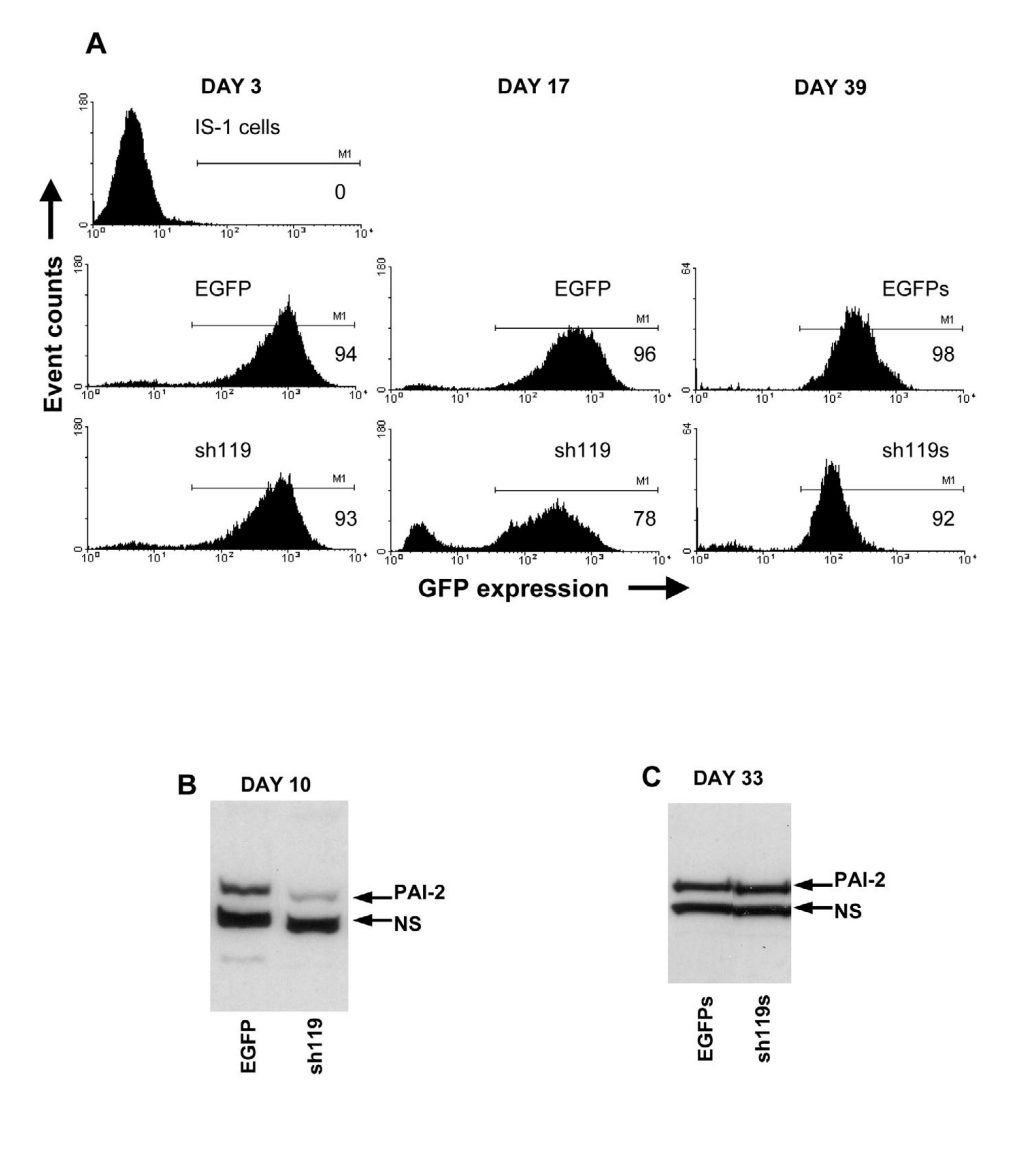
**Long-term gene silencing is not stable, despite persistent marker gene expression. **In A, IS-1 cells transduced with EGFP control or sh119 vectors were analysed by flow cytometry 3 and 17 days after transduction, and compared to non-transduced cells. EGFP expressing cells, from both transduced cell populations, were selected by cell sorting and named EGFPs and sh119s. 39 days after transduction, these cells were analysed for EGFP expression. Percentage EGFP positive cells, assessed by the M1 gating shown, are given in each histogram. In B, PAI-2 expression in EGFP and sh119 vector-transduced cell lysates were analysed by immunoblotting, 10 days after transduction. C is the same PAI-2 immunoblot as B, performed using EGFPs and sh119s cell lysates, 33 days after transduction.

In an attempt to generate a cell line with stable PAI-2 mRNA silencing without interferon response induction, we generated additional vectors which deliver 19 to 25 nucleotide shRNAs targeting different regions of the PAI-2 mRNA. Of these, the sh119 construct reduced PAI-2 expression, did not induce OAS1 and had no effect on cell morphology one week after transduction (data not shown). In parallel, we transduced IS-1 cells with the sh119 vector or a GFP control vector. We achieved high percentage transduction rates which were monitored for over two weeks (Figure 6A). GFP positive cells, from control- and sh119-transduced populations were sorted twice, using flow cytometry, to further enrich the GFP positive population of each (EGFPs and sh119s). Sh119-transduced cells showed PAI-2 gene silencing 10 days after transduction but not after one month of cell culture (Figure 6B and 6C). GFP marker gene expression was maintained in both sorted cell populations. During the prolonged culture period we noticed that, while the percentage of GFP positive cells remained stable, the intensity of GFP detected was reduced in the sh119-transduced cells, compared to GFP alone controls (Figure 6A). This was accompanied by a significant reduction in integrated vector copies (data not shown). Thus, although the marker protein was maintained at a reduced expression level, the prolonged culture period selected against cells with effective gene silencing. This suggests the presence of subtle cytotoxic effects of short dsRNA expression which are not apparent in the initial post transduction period and are in the absence of interferon response gene induction. Overexpression of PAI-2 in the IS-1 cells did not reduce the long-term selective effect on transduced cells or GFP expression levels in sh119-transduced cells (see Table 3).

**Table 3:** Percentage GFP positive cells and mean GFP fluorescence in sh119-transduced IS-1 and IS-1 PAI-2 cells. Transduced cells were assessed for GFP expression by flow cytometry. GFP positive cells were gated equally for each cell type, 5 and 31 days after transduction with the sh119 vector. Mean F is the mean fluorescence of gated GFP positive cells.

	% GFP positive cells		Mean F of GFP positive cells	
	
	Day5	Day31	Day5	Day31
	
IS-1 cells	85	40	146	83
IS-1 PAI-2 cells	82	46	180	96

## Discussion

Here we report the use of lentiviral vectors for the delivery of expression cassettes designed for RNAi-induced stable knock-down of gene expression. We undertook this approach because of the promise of RNAi to easily create cell lines that are specifically deficient in one protein component. By using lentiviral vectors for shRNA expression, high transduction efficiencies can be achieved, avoiding effects due to clonal selection of phenotypically different cells.

We successfully targeted the PAI-2 mRNA with the aim of studying the effects of reducing PAI-2 activity on cell sensitivity to apoptosis-inducing stimuli. PAI-2 activity has previously been implicated in protection from apoptosis [24]. The cytotoxic effect we observed, in cells transduced with RNAi-inducing vectors, appeared to correlate well with the reduction in PAI-2 protein levels. However, rapid selective growth pressure on cells bearing shRNA constructs with scrambled sequences, having no complementarity to the PAI-2 mRNA, suggested non-specific effects rather than a PAI-2-related phenotype. Using GFP as a marker gene, delivered by all vectors, enabled very sensitive detection of selective effects on transduced cells even when initial cell culture suggested stable transduction and cell growth.

We were able to detect increased expression of an interferon response gene, OAS1, in cells transduced with all hairpins of 21 or more base-paired nucleotides. A 21 mer hairpin induced the most potent OAS1 induction. These results, and those of others [21,22], suggest that dsRNAs of less than 30 nucleotides can induce an interferon response, even if they cannot directly activate protein kinase R [19]. The absence of OAS1 induction in cells transduced with 19 mer shRNAs implies that the search for appropriate hairpin sequences should be limited to stretches of this length or less.

Overexpression of functional PAI-2 did not rescue the cytotoxic effects or OAS1 induction observed in cells transduced with a series of vectors for expression of different length shRNAs. This result, the cytotoxicity associated with scrambled sequence hairpin-encoding constructs, and the same selective pressure seen on the growth of transduced HeLa cells, which do not express detectable PAI-2 mRNA or protein (Table 2 and data not shown), lead us to conclude that the phenotype we have seen in IS-1 cells is not PAI-2-specific. Without the ability to track transduced cells, via GFP expression, this conclusion would have been more difficult to obtain. In cells engineered to overexpress functional PAI-2, our RNAi-inducing vectors clearly reduced PAI-2 protein levels but did not significantly reduce the overexpressed PAI-2 mRNA. This suggests that in the presence of high concentrations of targeted mRNA, the machinery necessary for RNAi-induced mRNA cleavage is saturated and mRNA down-regulation undetectable. As protein levels are nevertheless reduced, the shRNA may be functioning post-transcriptionally, perhaps in a similar manner to natural miRNA. This phenomenon has been described elsewhere for siRNAs [27].

IS-1 cell transduction, using one PAI-2 targeting vector (sh119), initially appeared stable, compared to control-transduced cells. PAI-2 protein levels were clearly reduced 10 days after transduction and selective pressure on cell growth appeared to be minimal, as the percentage of sh119 GFP positive cells was apparently stable at about 80 %, 17 days after transduction. However, after cell sorting of GFP positive cells and prolonged cell culture of over one month after transduction, the PAI-2 antigen measured in sh119s (s for selected) cells was restored to control-transduced cell levels. Despite maintenance of transduction marker expression, gene silencing was absent. The GFP expression levels in selected sh119s cells was reduced after one month of growth, compared to cells monitored three days after transduction. It is possible that we selected cells with a greatly reduced, non-cytotoxic shRNA expression level, as we have detected a reduced number of integrated vector copies. The negative selective effect on cells transduced with the sh119 vector was not due to the suppression of PAI-2 expression, as PAI-2 overexpressing cells showed the same negative selection.

In all experiments in which a selective pressure on growth was apparent on shRNA-expressing cells, reduced percentage GFP positive cells and reduced GFP expression in transduced cells was measured over time. We hypothesised that very high expression levels of the various shRNAs is cytotoxic. This could occur via high numbers of transcriptionally active vector integration events or integration at transcriptionally active chromosomal regions. Both might be controlled using a tightly regulatable expression system, which has been described [15], but may require careful dosage in a gene- and cell-specific manner.

Our data demonstrate the importance of appropriate controls for using RNAi, as proposed in a recent editorial [28]. These include suppression of the RNAi phenotype by target gene overexpression, use of scrambled dsRNAs, and monitoring of non-specific gene expression in particular of interferon-responsive genes such as OAS1. Also, if considering the use of stable RNAi, it is imperative that stable knock-down is demonstrated as well as stable marker gene expression. In the cell culture system we used, conclusions drawn from experiments several days after RNAi delivery mask effects which are only apparent days later by monitoring the percentage of transduced cells. Such effects are likely be present in experiments using regulatable RNAi systems or using exogenously added dsRNA, where the experimental data linked to gene targeting may be collected before other effects are seen. The molecular events which lead to the long-term effect we have documented may well be underway during this experimental period.

In the light of our own data and other recent reports [20-22], solutions for the induced cytotoxic effects we describe here include testing a series of target sequences, using dsRNA of no more than 19 nucleotides at low effective vector doses, and careful monitoring of transduced cell phenotype with and without functional target gene overexpression. Long-term monitoring of gene silencing appears to be necessary in stable systems, even in the presence of marker gene expression.

## Conclusions

Our study demonstrates vector-derived RNAi in tumor cell lines and points towards the necessity of careful, but clearly feasible, controls when using RNAi for stable gene suppression in short- and long-term experiments.

## Methods

### Cell lines

Isreco-1 (IS-1) cells were a gift from Dr. B. Sordat (ISREC, Lausanne). 293T cells were a gift from Dr. D. Trono (Geneva University Medical Centre). HeLa cells were purchased from the European collection of cell cultures, ECACC number: 93021013. All cells were maintained in DMEM supplemented with 10 % Fetal Bovine Serum and 10 mM HEPES pH 7.4 (IS-1 medium) (purchased from Invitrogen).

### Plasmid constructions

Gene transfer plasmids, for RNA interference using lentiviral vectors, were constructed using the backbone of ploxEWiresGFP, a gift from Dr. P. Salmon, Geneva University Medical Centre. The human U6 gene was amplified by PCR using HeLa cell genomic DNA as template and the oligonucleotides U6-ClaI-F 5' GATC ATCGATAAGGTCGGGCAGGAAGAGGGCCTATTTCCC 3' and U6-ClaI-R 5' GATCATCGATTGGTAAACCGTGCACCGGCGATAAACG 3'. The 483 base pair PCR product was digested with ClaI, inserted into the ClaI site of pTRE2 hyg (Clontech), and its sequence verified by DNA sequencing. The U6 promoter and gene sequence corresponded to nucleotides 65 to 527 of Genbank accession number M14486. This plasmid was used as template for PCR reactions to amplify U6 promoter-driven expression cassettes. Each PCR product included the U6 promoter with shRNA-encoding sequences beginning at the U6 +1 site, and a run of 6 or 7 thymidine bases for an RNA polymerase III transcription termination signal. Each PCR introduced flanking ClaI restriction sites. PCR products were directly cloned into the pGEM Teasy (Promega) plasmid and sequenced. ClaI fragments of positive clones were excised and ligated into the unique ClaI site of ploxEWiresGFP. The U6 promoter cassettes in the resulting lentiviral vector plasmids were verified by DNA sequencing. Each construct used for vector production had the same orientation of the U6 expression cassette with respect to the *P*EF-1α-iresGFP region of the plasmid. A schematic of the gene transfer cassette is given in Figure 1A. Details of shRNA sequences used for each construct are given in Table 1. The gene transfer plasmid for PAI-2 overexpression was constructed by replacing the EGFP gene from ploxCW-GFP (a gift from Dr. P. Salmon, Geneva University Medical Centre) with the type B human PAI-2 (SERPINB2) [29] open reading frame.

### Vector production and transduction

Lentiviral vectors were produced by three plasmid co-transfection of 293T cells, essentially as described previously [30]. Vectors were harvested 48 hours after transfection, passed through 0.45 μm filters and used directly on target cells in a 1:1 ratio with IS-1 medium in a total volume of 2 ml. Transductions were performed in 3 cm diameter 6-well plates, on cells seeded the previous day at 5 × 10^4 ^or 1 × 10^5 ^cells/well. Upon addition of vectors, plates were centrifuged for one hour at 1000 g in the presence of 8 μg/ml polybrene (hexadimethrine bromide, Sigma). After 24 hours, target cells were washed twice with PBS and cultured in IS-1 medium until analysis. Where indicated, viral titre was determined by transducing cells with 10 μl of lentiviral vector conditioned medium from 293T cell producer cells and measuring the % of GFP positive cells by flow cytometry. We routinely achieve 10^6 ^effective transducing units per ml of producer cell conditioned medium, which results in a typical multiplicity of transduction of 10. Comparisons between cell populations transduced with different vectors is made by flow cytometry analysis of GFP positive cells.

### Antibodies

Anti-human PAI-2 monoclonal antibody 3750 was purchased from American Diagnostica. PE-labelled goat anti-mouse antibodies were purchased from Pharmingen. HRP-conjugated goat anti-mouse antibodies were purchased from Bio-Rad.

### Flow cytometry

Flow cytometry was performed using Becton Dickinson FACScan, FACStrack or FACScalibur instruments at the Geneva University Medical Centre flow cytometry facility. GFP was detected in cells detached and resuspended in FACS buffer comprising 1 % BSA in PBS supplemented with 0.05 % sodium azide. For the detection of intracellular PAI-2, a method adopted from Dr. M. Ranson (University of Wollongong, Australia) was used. Cells were detached and fixed in 0.25 % paraformaldehyde (PAF)/PBS for one hour on ice. Cells were permeabilised in 0.1 % saponin/PBS for 30 minutes at room temperature. Fixed, permeabilised cells were incubated for 30 minutes in PBS/0.5 % BSA/0.1 % saponin containing 2 μg/ml 3750 anti-PAI-2 monoclonal antibody, washed twice in PBS/0.1 % saponin and incubated for 30 minutes in 0.1 % saponin/0.5 % BSA/PBS/goat anti-mouse-PE antibodies. Finally, cells were washed twice in 0.1% saponin/PBS, twice in PBS and resuspended in 2.5 % PAF for flow cytometric analysis.

Sorting of live GFP positive cells was performed using a FACStar+ instrument (Becton Dickinson).

### Western blotting of cell lysates

Cells in suspension were lysed in 10 mM Tris-HCl (pH 7.4), 10 mM NaCl, 0.5 % NP-40, 3 mM MgCl_2_, 5 mM DTT and 1 mM PMSF for one hour on ice. Lysates were centrifuged at 16000 g for 5–10 minutes to remove nuclei and precipitates. Supernatant protein concentrations were measured using the Bio-Rad protein assay with BSA in lysis buffer as a standard. Cell lysates were separated by reducing SDS-PAGE and transferred to nitrocellulose membranes. Equal total protein lysate was used for each blot, between 2.5 and 10 μg depending on the assay. Membranes were blocked for 1 hour at room temperature in TBS-0.1 % Tween 20/5 % non-fat milk, and probed using antibodies in TBS-0.1 % Tween 20/5 % non-fat milk. The 3750 anti-PAI-2 antibody was used at a concentration of 1 μg/ml.

### Microscopy

Phase contrast microscopy was performed using a Zeiss Axiovert 100 M instrument on live cells. Images were collected using a Hamamatsu CCD camera (ORCA-100).

### PAI-2 functional activity

To measure PAI-2 functional activity, IS-1 and IS-1 PAI-2 cells were lysed in 1 % NP-40, 150 mM NaCl and 50 mM Tris pH 8.0. Thereafter, 6 μg of cleared total cell lysate was incubated with and without 10 U of low molecular weight urokinase (u-PA). 3 μg total cell lysate samples were then subjected to reducing SDS-PAGE and immunoblotting as for other PAI-2 immunoblots.

### Quantitative RT-PCR analysis of mRNA

Total RNA was isolated from cells using RNA preparation kits from Qiagen or TRIZOL^® ^reagent (Invitrogen). cDNA was generated using ImpromII^® ^reverse transcriptase (Promega) and random hexamers, according to the manufacturers instructions, typically using 1 μg of total RNA per reaction. Quantitative PCR was performed using an Applied Biosystems Prism 7000 instrument using Applied Biosystems SYBR^® ^green master mix reagent and oligonucleotide pairs to detect hypoxanthine phosphoribosyl transferase (HPRT), PAI-2 and oligoadenylate synthase-1 (OAS1) cDNA. 5' to 3' primer sequences were as follows: HPRT forward TATTGTAATGACCAGTCAACAG, HPRT reverse GGTCCTTTTCACCA GCAAG, PAI-2 forward GGGTCAAGACTCAAACCAAAG, PAI-2 reverse CCTTTGAAGTAGACAGCATTC, OAS1 forward AGGTGGTAAAGGGT GGCTCC and OAS1 reverse ACAACCAGGTCAGCGTCAGAT. Data were analysed using Applied Biosystems Prism software and the ΔC_T _method. Briefly, target gene expression was normalised to the HPRT endogenous reference gene for each sample. The difference between mean threshold PCR cycle values for target and control genes gave the ΔC_T _value. This was then calibrated to the control sample in each experiment to give the ΔΔC_T_ value, where the control had a ΔΔC_T _value of 0. The fold target gene expression, compared to the calibrator value, is given by the formula 2^-ΔΔCT^. Error bars represent the standard deviation of each target gene value, after evaluating the expression 2^-ΔΔCT+s ^and 2^-ΔΔCT-s^, where s is the standard deviation of the ΔΔC_T _value. All reactions were performed in duplicates or triplicates.

### ShRNA detection

Expression of shRNAs was detected using the *mir*Vana™ Probe Construction and miRNA Detection kits from Ambion (Austin), according to the manufacturers instructions. These kits employ in vitro transcription for radiolabelled probe generation and an RNase protection protocol for detection of small RNA expression, respectively. Briefly, radiolabelled RNA probes incorporating α-^32^P-UTP (Amersham) were constructed using T7 polymerase-driven transcription templates. Templates were designed to generate RNAs which hybridize to the endogenous Mir-16 miRNA, as a control, or the first 19 nucleotides of the sh325 shRNA (see table 1). The sh3 probe should therefore hybridize to any of the sh319/sh321/sh323 or sh325-derived shRNAs. Before hybridization, radiolabelled probes were purified by migration of the transcription reaction on a 12 or 15% Acrylamide/8 M Urea denaturing gel and elution of radioactive probe bands after detection by autoradiography. 3 μg of total cellular RNA was used for all hybridizations and RNase protections, supplemented with 2 μg yeast RNA as a carrier. Control reactions without target RNA but with or without RNase digestion included 5 μg yeast RNA. After hybridization and RNase digestion, protected probes were detected by autoradiography after migration in a 15% acrylamide/8 M Urea denaturing gel.

## Authors' contributions

RF carried out all of the experiments in this study and contributed to its conception, design and description. EKOK conceived the study and participated in its design and description. Both authors approved the final manuscript.
